# Molecular Networking and Mass2Motif Analysis Prioritise Glycosylated Triterpenoid-Related Chemical Space in the Cytotoxic Fractions of Edible *Sesbania grandiflora* Flowers

**DOI:** 10.3390/foods15193455

**Published:** 2026-09-27

**Authors:** Thanh-Sang Nguyen, Hieu T. Le, Joo Young Kim, Dieu T. X. Nguyen

**Affiliations:** 1 Faculty of Food Technology, Saigon Technology University, Ho Chi Minh City 700000, Vietnam; sang.nguyenthanh@stu.edu.vn; 2 Department of Pharmacognosy and Traditional Pharmacy, School of Pharmacy, University of Medicine and Pharmacy at Ho Chi Minh City, Ho Chi Minh City 700000, Vietnam; trunghieu2393@gmail.com; 3 Department of Pharmacology, College of Medicine, Yonsei University, Seoul 03080, Republic of Korea; jooyoungkim@yuhs.ac

**Keywords:** *Sesbania grandiflora*, edible flowers, feature-based molecular networking, MS2LDA, Mass2Motif, triterpenoid saponins, bioactivity-guided fractionation

## Abstract

*Sesbania grandiflora* flowers are widely consumed as vegetables in several South and Southeast Asian countries, but the chemical constituents associated with their reported cytotoxicity remain poorly defined. An ethanolic flower extract was separated into four solvent fractions, profiled by UHPLC-QTOF-MS/MS, and evaluated against A549 and HepG2 cells. Cytotoxicity was concentrated in the ethyl acetate and *n*-butanol fractions, with IC_50_ values ranging from 27.7 to 42.4 µg/mL. Among 3015 aligned features, 187 showed abundance patterns that tracked with the active fractions. Terpenoids accounted for 121 of these features, representing 64.7% of the activity-tracking set compared to 31.9% of the whole feature set, indicating a disproportionate representation of terpenoid-related chemistry. Eighteen molecular families were prioritised by family-level enrichment analysis, of which 13 were dominated by terpenoids. Complementary MS2LDA analysis identified nine enriched Mass2Motifs, including six triterpenoid-related motifs, two flavonoid-related motifs, and one structurally unresolved motif. A total of 334 features were supported by both family- and substructure-level analyses, with 80.5% classified as terpenoids. These enrichment analyses were exploratory and used for prioritisation rather than as confirmatory statistical tests. Chromatographic separation of the ethyl acetate fraction yielded nine NMR-confirmed constituents, including oleanolic acid, kaempferol, and 3-O-α-L-rhamnopyranosyl-(1→3)-β-D-glucuronopyranosyl oleanolic acid (SGE20). SGE20 was the most active tested isolate, with IC_50_ values of 33.08 and 47.54 µM against A549 and HepG2 cells. The corresponding feature belonged to an enriched molecular family and carried enriched Mass2Motifs, thereby placing the most active tested isolate within the chemical region defined by the family- and substructure-level analyses. Overall, the combined biological and metabolomic analyses consistently prioritised glycosylated triterpenoid-related chemistry, together with a smaller flavonoid component, as the principal chemical region co-distributed with fraction-level MTT activity. Given the established dietary use of *S. grandiflora* flowers, these findings provide baseline chemical information for future studies examining how culinary processing and realistic dietary exposure affect the prioritised constituent families.

## 1. Introduction

*Sesbania* is a pantropical legume genus whose species are used for food, fodder, timber, soil improvement, and traditional medicine [1]. *Sesbania grandiflora* is among the most widely used members of the genus, and its flowers and young pods are commonly cooked and consumed as vegetables in Vietnam, Thailand, Indonesia, India, and Sri Lanka. The flowers are also reported to be sources of vitamin C and calcium [1,2]. This long-standing dietary use makes their chemical composition relevant not only to natural-product research but also to the evaluation of biologically active constituents in an edible plant. Chemically, *S. grandiflora* has been reported to contain terpenoids, phenylpropanoids, isoflavonoids, alkaloids, saponins, sterols, and amide lipids, while extracts from different plant parts have shown cardioprotective, antidiabetic, analgesic, anti-inflammatory, antimicrobial, and cytotoxic activities [1,2].

Among these reported activities, cytotoxicity has been studied extensively. Sesbanimide-type alkaloids, which are potent cytotoxins, occur in the genus [2]. Benzofurans isolated from the stems of *S. grandiflora*, including spinosan A, spinosan B, and sesbagrandiflorain C, inhibit tumour cell growth [3,4]. Ethanolic leaf and flower extracts suppress tumour growth in Ehrlich ascites carcinoma-bearing mice, and a protein fraction from the flowers (SF2) induces caspase-dependent apoptosis in several cell lines [5,6,7]. Triterpenic acids and their glycosides from *S. sesban* have also shown antiproliferative activity [8]. Beyond cytotoxicity, a flavonoid-rich fraction of *S. grandiflora* has been reported to be hepatoprotective in vivo and cytoprotective in HepG2 cells [9], while phenolic extracts show antioxidant and anti-fibrotic effects [10]. Root constituents including isovestitol, medicarpin, sativan and betulinic acid are active against *Mycobacterium tuberculosis* H37Rv [11], and cardioprotective and antihyperglycaemic effects have also been described [12,13].

Despite this broad biological record, the relationship between the observed activities and the underlying chemical composition remains poorly resolved. Most previous studies have either evaluated whole extracts or focused on individual compounds obtained after repeated chromatographic isolation. Whole-extract assays provide limited information on which constituents contribute to activity, whereas conventional bioactivity-guided isolation can favour abundant and readily purified compounds while overlooking minor constituents, unstable metabolites or groups of structurally related compounds. Untargeted LC-MS/MS, combined with feature-based molecular networking (FBMN), provides an alternative strategy by organising the detected metabolome into molecular families according to MS/MS spectral similarity before isolation [14]. These molecular families can then be examined in relation to fraction-level bioactivity to prioritise chemically coherent regions of the metabolome for further investigation.

An additional challenge is that solvent-fractionation experiments usually contain only a small number of fractions, while untargeted metabolomics can generate thousands of features. Under these conditions, strong feature-level correlations can arise from very limited numbers of observations and should therefore be interpreted cautiously. In the present study, feature-level activity tracking was used primarily as a prioritisation step, while greater emphasis was placed on patterns reproduced across molecular families and recurring MS/MS substructures. This strategy was intended to reduce reliance on individual feature correlations and to identify broader chemical patterns associated with the active fractions.

This study aimed to identify metabolite features, molecular families, and recurring MS/MS substructures that co-varied with fraction-level reductions in MTT viability and to evaluate whether isolation of selected constituents supported this prioritisation. Selected constituents of the ethyl acetate fraction were subsequently isolated and structurally characterised to examine how far the metabolomics-based prioritisation could be supported experimentally. Given the established use of *S. grandiflora* flowers as a vegetable, these findings also provide a basis for future studies examining how culinary processing may influence the occurrence and biological relevance of the identified constituent families.

## 2. Materials and Methods

### 2.1. Plant Material

Fresh inflorescences of *S. grandiflora* were collected in July 2024 in Ben Tre Province, Vietnam (10.15° N, 106.58° E), and authenticated by Dr. Dieu T. X. Nguyen. A voucher specimen (SG_BT_2407) is held at the Department of Pharmacognosy and Traditional Pharmacy, School of Pharmacy, University of Medicine and Pharmacy at Ho Chi Minh City. The material (32 kg fresh weight) was air-dried in the shade at room temperature and milled, giving about 3.0 kg of coarse powder, which was kept in airtight containers at ambient temperature until extraction.

### 2.2. Chemicals, Reagents and Instruments

Solvents were from Thermo Fisher Scientific (Seoul, Republic of Korea). Methanol and acetonitrile for UHPLC-HRMS were of Optimal^TM^ LC/MS grade; solvents for extraction and isolation were of analytical grade. Deuterated NMR solvents containing 0.03% tetramethylsilane were from Euriso-Top (Saint Aubin, France). TLC used silica gel 60 F254 aluminium plates (Merck, Darmstadt, Germany). Column chromatography used Sephadex LH-20 (Pharmacia Biotech, Uppsala, Sweden), Toyopearl HW-40C (Tosoh Bioscience, Tokyo, Japan), silica gel (40–63 µm and 15–40 µm; Merck, Darmstadt, Germany), and RP-18 (Merck, Darmstadt, Germany). NMR spectra were recorded on a Bruker AM500 (Bruker, Rheinstetten, Germany; 500 MHz for ^1^H, 125 MHz for ^13^C). UHPLC-HRMS used a Waters ACQUITY UPLC H-Class coupled to a Xevo G2-XS QTOF (Waters Corporation, Milford, MA, USA).

### 2.3. Extraction and Fractionation

Dried flower powder (3.0 kg) was percolated with 96% ethanol (90 L) over 3 consecutive days at room temperature and protected from direct light. The combined percolates were concentrated under reduced pressure at a temperature not exceeding 40 °C to yield 780 g of crude extract (SG), corresponding to an extraction yield of 26.0% (*w*/*w*, based on dried flower material). A portion of SG was retained as a reference sample, and the remainder was suspended in water and partitioned successively against solvents of increasing polarity to give *n*-hexane (SGH, 98 g), ethyl acetate (SGE, 130 g), *n*-butanol (SGB, 210 g), and aqueous (SGW, 288 g) fractions. Relative to the initial crude extract mass, the recovered fraction masses corresponded to 12.6%, 16.7%, 26.9%, and 36.9%. These masses were equivalent to yields of 3.27%, 4.33%, 7.00%, and 9.60% based on the initial dried flower material. The fractions were dried to constant weight in a vacuum oven, and portions of the same dried samples were used for the MTT assay and molecular networking analysis. All materials were dried and stored at −20 °C.

### 2.4. UHPLC-HRMS/MS

UHPLC-HRMS analysis was performed on a Waters ACQUITY UPLC H-Class system coupled to a Xevo G2-XS QTOF mass spectrometer. Separation used a Waters ACQUITY UPLC BEH C18 column (2.1 × 50 mm, 1.7 µm) with water (A) and acetonitrile (B), both containing 0.1% formic acid, at 0.3 mL/min and 40 °C. Solvent B rose linearly from 10% to 100% over 12 min, was held for 3 min, and then returned to 10% for a 3 min re-equilibration (15 min total). The injection volume was 2 µL.

Detection was determined by ESI in positive mode with a QTOF analyser in MS^E^ mode. Capillary and sample cone voltages were 3.0 kV and 40 V, source and desolvation temperatures were 100 °C and 250 °C, and cone and desolvation gas flows were 60 and 1200 L/h. Data were acquired from *m*/*z* 100 to 1500 with a 0.2 s scan time, at a fixed collision energy of 6.0 eV in the low-energy function and a 20.0–40.0 eV ramp in the high-energy function. Mass accuracy was maintained with a LockSpray using leucine enkephalin (cone 30 V, capillary 3.0 kV, DRE transmission 99.9%) at 10 s intervals with a 0.25 s scan time, and the instrument was calibrated with sodium formate before each sequence. Samples were dissolved in methanol at 0.1 mg/mL and filtered through 0.22 µm membranes. Each fraction was injected twice as technical replicates of the same prepared sample solution, and a methanol blank was included in the sequence.

Because MS^E^ acquires fragment spectra without physical precursor isolation, precursor–fragment relationships were reconstructed in MS-DIAL using retention-time deconvolution with the MS2Dec module and a sigma window of 0.5. Peak detection used a minimum height of 1000 a.u.; fragment ions were kept above an absolute abundance of 100 and a relative abundance of 1%; isotope ions of the precursor were excluded from the reconstructed spectra. Reconstructed spectra contained 20–80 fragment ions and were exported as MGF for downstream analysis.

### 2.5. Data Processing and Annotation

Raw data were centroided in MassLynx and processed in MS-DIAL v5.5.250627 [15]. Tolerances were 0.01 Da for MS1 and 0.05 Da for MS2. Peaks were picked at a minimum height of 1000 a.u. with a 0.1 Da mass slice; smoothing was performed using a linear weighted moving average over three scans with a minimum peak width of five scans, and alignment used a retention-time tolerance of 0.1 min. Adducts considered were [M+H]^+^, [M+NH_4_]^+^, [M+Na]^+^, [M+K]^+^, [M+H−H_2_O]^+^, [M+H−2H_2_O]^+^, [2M+H]^+^, [2M+NH_4_]^+^, and [2M+K]^+^. Isotopes and multiply charged species were constrained during peak detection to a maximum of two isotopes and a maximum charge state of 2. Features were removed on the basis of the methanol blank using a sample-maximum-to-blank-average fold-change criterion of ≥5, calculated separately for each sample group so that fraction-specific ions were not diluted against the blank. A feature was retained if detected in at least half of the injections of at least one group, and remaining gaps were recovered by compulsory gap filling. Alignment yielded 3015 features, of which 2924 carried a usable MS/MS spectrum.

Compound annotation and chemical classification were performed as related but distinct steps. Compound-level annotation was based on spectral matching in MS-DIAL using MSMS_Public_EXP_Pos_VS17, MSMS_Public_ExpBioInsilico_Pos_VS17, MoNA LC-MS/MS Positive Mode, GNPS, and Spectraverse v1.01. An in-house taxonomic candidate list of 81 Sesbania natural products previously reported in the literature, compiled from Reaxys as chemical structures without associated references or in silico spectra, was additionally used to support annotation of compounds relevant to the genus. For broader chemical classification, the exported MS/MS spectra were processed through an R-based workflow integrating spectral information with NPClassifier-based structural classification [16,17,18]. The workflow assigned pathway, superclass, and class information where sufficient spectral or annotation support was available and propagated broader class information among spectrally related features when supported by the corresponding molecular family. These assignments were used to describe chemical composition at the class level and were kept distinct from specific compound annotations. Annotation confidence followed Schymanski et al. [19], with modifications according to Nguyen et al. (2026) [20]. Compounds confirmed by HR-MS and NMR were assigned as Level 1. Features supported by experimental spectral-library matching were assigned as Level 2, whereas assignments supported only at broader structural or chemical class levels were treated as Level 3. NPClassifier pathway, superclass, and class assignments were therefore used as chemical classifications rather than evidence of specific compound identities.

### 2.6. Molecular Networking

Spectral similarity networks were computed in R with matchms [21] using three scoring functions: modified cosine, Spec2Vec [22], and MS2DeepScore [23]. Edges were kept for the mutual top-10 neighbours of each feature above 0.7 (modified cosine, Spec2Vec) or 0.8 (MS2DeepScore). The three networks were compared and combined into a consensus network retaining edges recovered by at least two of the three methods.

### 2.7. Substructure Discovery

Unsupervised substructure discovery used MS2LDA [24,25] on the same MGF export. Fragment and neutral-loss features were binned at 0.01 Da, and 300 Mass2Motifs were inferred by latent Dirichlet allocation. A feature was assigned to a motif when it passed both the probability and overlap thresholds recommended for MS2LDA (0.3 and 0.3).

### 2.8. Cell Lines and Cytotoxicity

A549 (human lung carcinoma) and HepG2 (human hepatocellular carcinoma) cells were kindly provided by Prof. J. M. Pezzuto (Long Island University, Brooklyn, NY, USA) and Prof. Chi-Ying Huang (National Yang Ming Chiao Tung University, Taipei, Taiwan). HepG2 was authenticated by short tandem repeat (STR) profiling at the Institute of Genome Research, Vietnam Academy of Science and Technology; an equivalent STR record was not available for A549. Both cell lines were routinely screened for mycoplasma contamination (MycoStrip^®^ 2.0, InvivoGen, San Diego, CA, USA) and tested negative. No non-cancerous cell line was included, so selectivity indices were not calculated and the results below should not be read as evidence of selectivity for tumour cells.

Cytotoxicity was measured by MTT assay. Test solutions were prepared in DMSO and serially diluted in FBS-free medium. Extracts and fractions were tested at six final concentrations of 0.8, 4, 20, 30, 50, and 100 µg/mL, whereas pure compounds were tested at six final concentrations of 0.4, 2, 10, 50, 100, and 200 µg/mL. For pure compounds, the corresponding molar concentrations and IC_50_ values in µM were subsequently calculated using their respective molecular weights. Cells were trypsinised, counted on a haemocytometer and seeded in 96-well plates in 190 µL of medium containing 5% FBS. After 18–20 h at 37 °C, 10 µL of test solution was added per well. Negative controls received 190 µL medium plus 10 µL of 1% DMSO and blanks contained the medium only. Ellipticine served as a positive control. After 48 h, 10 µL of MTT was added to a final concentration of 0.5 mg/mL, and after a further 4 h, the medium was removed and the formazan dissolved in 50 µL DMSO. Absorbance was read at 540 nm on a BioTek Elx800 reader. Growth inhibition was calculated relative to untreated controls, and IC50 values were fitted using TableCurve 2D v4.0 with Equation 8010, an empirical three-parameter power function (percentage inhibition = a + b × concentration^c^), with parameters estimated by unweighted least squares. IC_50_ was defined as the concentration corresponding to 50% inhibition on the fitted curve. As a confirmatory analysis, concentration–response data were independently fitted using a two-parameter logistic model with fixed 0% and 100% plateaus, and 95% confidence intervals for IC_50_ were estimated by parametric bootstrap (1000 resamples). Each concentration was tested in three replicate wells within a single experiment (*n* = 3 technical replicates).

### 2.9. Activity Association, Feature Prioritisation and Family-Level Enrichment

Because the analysis included only four solvent fractions, the Spearman statistic was used solely as a rank-order filter and was not interpreted as inferential evidence of an activity-feature correlation. Cytotoxicity values were converted to a potency scale by taking the negative logarithm of the IC_50_ value in µg/mL, so that higher values represented greater potency. Fractions that did not reach 50% inhibition at the highest concentration tested were assigned the censoring limit of 100 µg/mL before transformation. Feature intensities were analysed as raw peak areas because all fractions were injected at the same mass concentration. Association between feature intensity and potency across the four fractions was assessed using Spearman rank correlation. Ties, including the two fractions censored at the same imputed value, were assigned average ranks, and with only four fractions ***ρ*** could take only a limited number of discrete values, so that the smallest attainable Benjamini–Hochberg-adjusted *p* value at the individual feature level was far above conventional significance thresholds. The effect size between the active and inactive fractions was calculated as

log_2_FC = log_2_[(mean intensity in active fractions + 1)/(mean intensity in inactive fractions + 1)] (1)

A feature was designated as activity associated when Spearman ρ ≥ 0.8 and log_2_FC ≥ 2, corresponding to at least four-fold enrichment in the active fractions. Benjamini–Hochberg procedures were used to adjust correlation *p* values for multiple testing. A composite score was used to rank features for visualisation

Feature score = 0.7 × max(ρ, 0) + 0.3 × min[max(log_2_FC, 0)/FC_95_, 1] (2)

where FC_95_ is the 95th percentile of positive log_2_FC values and was used to reduce the influence of extreme fold changes. Features were then grouped according to their connected components in the consensus molecular network, with unconnected features treated as components of size one. Each component received

Cluster score = median(feature score) × f × [1 + ln(min(n, 30))/ln(10)](3)

where f is the proportion of component members with ρ ≥ 0.8, and n is the component size. The component size was capped at 30 to prevent large clusters from being excessively weighted. The final priority score for each feature was calculated as

Priority score = √(feature score × cluster score)(4)

The feature, cluster, and priority scores were used only for ranking and visualisation and were not interpreted as statistical tests. Enrichment was evaluated separately for molecular families, Mass2Motifs, and chemical classes. For molecular families, feature labels were permuted 10,000 times while network membership was kept fixed. Two statistics were recalculated for each permutation, the mean log_2_FC of family members and the proportion of members classified as activity associated. Empirical *p* values were calculated as

*p* = [1 + #(permuted statistic ≥ observed statistic)]/10,001 (5)

and corrected using the Benjamini–Hochberg procedure [26] across molecular families containing at least five members. Mass2Motif enrichment was assessed using a one-sided Fisher exact test for motifs containing at least three members, followed by the same multiple testing correction. Chemical class enrichment was tested in the same manner against the complete detected feature set. The consensus molecular network and MS2LDA model were constructed from MS/MS data independently of the cytotoxicity measurements.

### 2.10. Isolation and Structure Elucidation

The fraction with the strongest cytotoxicity was taken forward for phytochemical work. Constituents were separated by repeated normal-phase silica gel column chromatography and gel permeation on Sephadex LH-20. Structures were determined from HR-MS together with one- and two-dimensional NMR and by comparison with previously reported spectroscopic data.

### 2.11. Software and Statistics

Data handling, network construction, permutation testing, enrichment analysis, and figure preparation were performed in R v4.4 under RStudio v2024.12.0+467. Permutation and enrichment analyses were exploratory and used for candidate prioritisation rather than confirmatory statistical inference.

## 3. Results and Discussion

### 3.1. Chemical Profile of the Fractions and Annotation Coverage

The analytical and computational workflow is summarised in Figure 1. Alignment of the four fractions gave 3015 features, 2924 of which carried a usable MS/MS spectrum. Compound-level annotation coverage was relatively low, as commonly encountered in untargeted analyses of plant extracts from under-studied taxa. Based on experimental spectral matching, the in-house taxonomic candidate list, and additional computational evidence, 1064 of the 3015 features (35.3%) received at least one compound-level annotation, whereas 1951 features lacked a specific compound assignment. This level of coverage is expected rather than exceptional, since only a proportion of spectra acquired in untargeted metabolomics can be matched to reference spectra [27], and public libraries remain biassed towards commercially available compounds and extensively studied taxa [28]. Experimental spectral-library matches were reported at Level 2, whereas broader structural or chemical class assignments were treated as Level 3 [19,20]. Accordingly, the analyses below rely primarily on chemical classes and molecular families rather than on specific compound identities.

Chemical classification was evaluated separately from compound-level annotation. Using the MS/MS-based classification workflow described in Section 2.5, 1719 of the 3015 features (57.0%) could be assigned an NPClassifier pathway, while 1296 features (43.0%) remained unclassified at this level (Figure 2B). Thus, the greater coverage of pathway-level classification compared with specific compound annotation reflects the broader structural resolution of the classification workflow and should not be interpreted as additional compound-level identification. Among all the detected features, terpenoids formed the largest pathway, accounting for 961 features (31.9%), followed by shikimates and phenylpropanoids at 361 features (12.0%). Alkaloids, polyketides, fatty acids, amino acids, and peptides together accounted for less than 15% of the feature set. The same NPClassifier pathway framework applied to the 81 Sesbania compounds recorded in Reaxys produced a different distribution (Figure 2A). Terpenoids occupied a similar proportion of the literature record, whereas shikimates and phenylpropanoids accounted for approximately 30% of reported compounds compared with 12.0% of the detected features. Fatty acids were also proportionally more represented in the literature dataset. Part of this difference may be organ dependent, since the published chemistry of the genus derives largely from the roots, stems, and leaves, whereas the present dataset describes the flowers. It may also reflect biases associated with classical isolation, because aglycones and moderately polar phenolics are generally more tractable to purification and structural characterisation than complex polar glycoside series.

The larger proportion of unclassified features reflects the limitations of assigning broad chemical information from untargeted MS/MS data. Computational chemical classification can extend structural information beyond features with specific library matches, but its coverage remains dependent on the quality and informativeness of the available spectral evidence [16,17,18]. Features with weak or limited MS/MS information, high molecular mass, or uncertain charge states may therefore remain difficult to place confidently within a recognised chemical pathway. The unclassified group should not be interpreted as a distinct chemical group, but rather as features for which the available evidence was insufficient for pathway-level assignment. Consequently, subsequent analyses focused on chemical classes, molecular families defined by spectral similarity, and recurring substructures inferred from the MS/MS spectra rather than on individual compound identities.

### 3.2. Molecular Families and Diagnostic Fragmentation

The consensus spectral similarity network contained 2924 features with usable MS/MS spectra and 5090 edges. For the family-level summary, the full dataset of 3015 features was considered, with the 91 features lacking usable MS/MS spectra treated as singletons, giving a total of 852 molecular families or singleton groups (Figure 3, Table S5). Of these, 623 were singletons, representing 20.7% of all detected features, and 32 of the 187 activity-tracking features occurred in this category. A further 165 families contained two to four features, comprising 407 features (13.5% of the dataset) and 14 activity-tracking features. In contrast, 64 families contained at least five features and together accounted for 1985 features (65.8% of the dataset), including 141 of the 187 activity-tracking features. Thus, approximately two thirds of the detected metabolome occurred in molecular families of sufficient size for family-level permutation analysis, whereas the remaining features were either singletons or members of small families and were not included in this analysis.

Two dominant putative chemical patterns were apparent among the annotated molecular families. Triterpenoid aglycones and their glycosides, together with phytosterol glycosides, formed most of the larger annotated families. Flavonoid aglycones and their O glycosides formed smaller and more distinct families. This grouping is consistent with their fragmentation behaviour. Features sharing a similar aglycone-like fragment pattern and sugar-sized neutral losses commonly retain characteristic aglycone fragment ions while differing mainly in the neutral losses of their sugar residues. They can therefore cluster within the same molecular family while remaining separated from compounds with different core structures.

The flavonoid and triterpenoid families also showed distinct MS/MS patterns (Figure 4). In the flavonoid group, the aglycone ion at *m*/*z* 287.06 produced fragment ions at *m*/*z* 153.02, 165.02, and 213.06. The same *m*/*z* 287.06 ion was also prominent in spectra of the corresponding glycosylated features. Several members differed by 162.05 Da, consistent with the loss of a hexose residue. The predominance of the aglycone ion is consistent with the expected fragmentation of flavonoid O glycosides, in which cleavage of the glycosidic bond readily releases the aglycone [29,30]. Neutral losses of 146 Da and 308 Da, corresponding to deoxyhexose and rutinose, have also been reported for this class [29,30], although these losses were not observed among the features shown here. The triterpenoid family showed a different fragment pattern, with common ions at *m*/*z* 189.16, 191.18, 203.18, 205.20, and 249.19. These ions are consistent with fragmentation of a pentacyclic triterpenoid core. Additional ions at *m*/*z* 439.36, 437.34, and 455.35 were consistent with oleanane-related aglycones and their dehydration products [31,32]. Glycosylated members produced similar aglycone and core fragment ions, while signals from the intact precursor were weak. Their sugar composition therefore cannot be determined reliably from the fragment spectra alone and must instead be inferred cautiously from the mass differences between related features.

These fragmentation patterns support annotation mainly at the structural class level. The ion at *m*/*z* 287 is consistent with kaempferol, but other flavonoids with the same elemental composition can produce the same precursor mass and similar fragment ions. The ions at *m*/*z* 153, 165, and 213 are also shared by several flavonoid isomers [30]. A similar limitation applies to pentacyclic triterpenoids because oleanane, ursane, and lupane structures can generate overlapping fragment patterns [31,32]. The spectra can therefore support assignment to a pentacyclic triterpenoid class, but not reliable discrimination among individual skeletons. Neutral losses of 176, 162, 146, and 132 Da can indicate the presence of common sugar residues, but they do not establish anomeric configuration, glycosidic linkage position, or the attachment site on the aglycone. For this reason, MS/MS-based annotations are reported as tentative. Level 2 annotations are used when a library match supports a specific structure, whereas Level 3 annotations are used when only the structural class can be assigned [19]. Only compounds confirmed by NMR are treated as identified.

### 3.3. Cytotoxicity of the Fractions and Feature-Level Activity Association

The greatest reduction in MTT-derived cell viability was observed for the two intermediate-polarity fractions. SGE showed IC_50_ values of 30.44 µg/mL against A549 cells and 27.66 µg/mL against HepG2 cells, while SGB gave values of 31.17 and 42.44 µg/mL, respectively. In contrast, SGH and SGW showed IC_50_ values above 100 µg/mL against both cell lines (Table 1). Ellipticine showed substantially lower IC_50_ values than both active fractions. These results indicate moderate in vitro activity of SGE and SGB in the MTT assay under the tested conditions. The restriction of cytotoxicity to SGE and SGB suggests that chemical features co-distributed with the lower-IC50 fractions were preferentially detected in the ethyl acetate and *n*-butanol partitions Solvent partitioning separates compounds according to physicochemical properties and can enrich metabolites that are present at lower relative abundance in the original extract. The biological contrast between the two active fractions and the two inactive fractions therefore provided a basis for asking which detected metabolites followed the same distribution as cytotoxicity.

The metabolomic comparison was therefore used as an exploratory screen rather than as a feature-level significance test. A total of 187 features showed both a rank pattern consistent with the cytotoxicity profile and at least a four-fold higher abundance in SGE and SGB than in SGH and SGW (Tables S2 and S3). These features are referred to here as activity-tracking features because their distribution follows the fraction-level phenotype, but this does not establish that they contribute directly to cytotoxicity.

Bioactivity-based molecular networking was originally developed to combine metabolite abundance across chromatographic fractions with fraction-level bioactivity [33] and has subsequently been applied to guide the isolation of active natural products [34]. However, the limited number of fractions in the present study prevents reliable statistical inference at the level of individual features. With only four observations, Spearman correlation has low resolution and cannot achieve conventional significance after multiple testing. The correlation term was therefore used only to retain features whose abundance ordering matched the activity ordering, while the log2 fold change provided the main measure of enrichment between active and inactive fractions. Because A549 and HepG2 ranked the four fractions identically, both assays produced the same feature set and should not be treated as independent evidence. The 187 features were consequently taken forward as a candidate pool for family-level and substructure-level analyses, where enrichment could be tested against an appropriate null distribution.

Mapping the prioritised features onto the consensus molecular network provided a clearer chemical pattern (Figure 5). Features with high activity priority were concentrated in several molecular clusters rather than appearing as isolated nodes throughout the network. This organisation is relevant because the network itself was constructed from MS/MS spectral similarity without using either feature intensity or cytotoxicity [14,20,21,22]. Structurally related metabolites therefore formed the families before the biological data were considered. The subsequent concentration of activity-associated features within particular families suggests that the biological phenotype is associated with specific regions of chemical space. This observation does not identify the active compounds, but it provides a more informative level of prioritisation than individual feature correlations derived from only four fractions.

The chemical composition of the 187 features further narrowed this pattern (Figure 6). Terpenoids accounted for 121 features, corresponding to 64.7% of the activity-associated set. Shikimates and phenylpropanoids accounted for 18 features, followed by alkaloids, amino acids and peptides, polyketides, and fatty acids in much smaller numbers. Thirty-eight features remained unclassified. Within the terpenoid group, oleanane-related structures were prominent, together with smaller groups assigned to ursane, taraxastane, lupane, lanostane, cycloartane, and cucurbitane skeletons. Steroid-related cholestane and ergostane structures were also present. Many of these features occurred as putative glycosides of hederagenin, soyasapogenol, and related aglycones, consistent with the glycoside fragmentation patterns described in Section 3.2.

This chemical pattern is biologically plausible because triterpenoid and steroidal saponins from other plant species have shown cytotoxic effects in the same cellular models used here. Recent studies have reported cytotoxic triterpenoid saponins against A549 cells and steroidal saponins with activity against HepG2 and A549 cells [35,36]. These reports do not establish that the *S. grandiflora* features act through the same mechanisms, but they support the biological relevance of prioritising the saponin-rich families observed here. This literature comparison does not identify the observed *S. grandiflora* features as the same structures or establish a shared mechanism. The structural diversity within this group is also important. The activity-associated features do not represent one saponin series but several related triterpenoid and steroidal families, suggesting that the observed fraction-level phenotype may reflect a mixture of structurally related compounds rather than a single dominant metabolite. A possible biological contribution from membrane interactions should also be considered. Saponins contain a hydrophobic aglycone linked to one or more polar sugar residues, giving them properties that can influence lipid bilayers. Recent biophysical work with the triterpenoid saponin ginsenoside Rh2 showed direct interaction with membrane lipids and changes in lipid packing that could increase membrane permeability [37]. Such behaviour provides one possible explanation for cytotoxicity associated with saponin-rich fractions. It cannot, however, be inferred from the present MTT assay. Reduced MTT conversion indicates the loss of cellular metabolic activity but does not distinguish apoptosis, inhibition of proliferation, mitochondrial dysfunction, or loss of membrane integrity. Measurements such as LDH release, membrane permeability, Annexin V staining, caspase activation, and analysis of non-cancerous cells would be required to determine which of these processes contributes to the activities of SGE and SGB.

The network also contained a smaller but distinct flavonoid component. A family containing the kaempferol glycoside series described in Section 3.2 included several activity-associated members. This suggests that the phenotype associated chemistry is not exclusively terpenoid. Instead, the two active fractions contain at least two chemically distinct groups that follow the same biological distribution. This point becomes relevant in the subsequent isolation experiment, where kaempferol was recovered from SGE and matched an activity-associated LC-MS feature.

Family size also affected interpretation of the network. The largest molecular family contained 375 features and included 52 activity-associated features, but these represented only about 14% of the family. In contrast, several smaller families contained a much larger proportion of activity-associated members. A large number of associated features within a large family is therefore not necessarily evidence of a stronger relationship with cytotoxicity. The subsequent permutation analysis addresses this issue by testing whether the proportion of associated features or the mean fold change within each family is greater than expected from the background distribution.

An additional limitation is that structural coherence does not establish biological contribution. Compounds within the same molecular family often share not only structural features but also polarity and chromatographic behaviour. Related glycosides may therefore partition together into SGE or SGB regardless of whether each compound contributes to cytotoxicity. The 187 features should consequently be viewed as a chemically restricted candidate pool rather than a list of active metabolites. The same candidate region was represented consistently at two complementary levels of MS/MS organisation: whole-spectrum similarity and recurring substructures. For this reason, the following analyses test whether these features are enriched within specific molecular families and independently recovered as shared MS/MS substructures. Convergence between fraction-level activity, molecular family enrichment, and substructure enrichment provides a more defensible basis for selecting candidates for isolation than any individual feature derived from the four-fraction comparison.

### 3.4. Molecular Families and Substructures Enriched in Activity-Tracking Features

Consistent with Section 2.11, the family- and motif-level enrichment analyses reported below were exploratory and used for candidate prioritisation rather than as confirmatory statistical inference. The 187 activity-tracking features identified in Section 3.3 were first examined at the chemical class level to determine whether the distribution was biassed toward particular types of metabolites. Terpenoids accounted for 121 features, representing 64.7% of the activity-tracking set compared with 31.9% of all 3015 detected features. This corresponded to an odds ratio of 4.34 with a Fisher *p* value of 1.6 × 10^−21^ (Table 2). No other NPClassifier pathway was significantly enriched. Shikimates and phenylpropanoids accounted for 18 features, while alkaloids, amino acids and peptides, polyketides, and fatty acids occurred at far lower frequencies. Thirty-eight features remained unclassified at the pathway level. The enrichment therefore indicates that the chemistry concentrated in SGE and SGB was disproportionately terpenoid instead of simply reflecting the overall abundance of terpenoids in the flower metabolome.

Classification at finer structural levels showed that this signal was mainly associated with triterpenoid chemistry. Of the 187 activity-tracking features, 68 could be assigned at the NPClassifier superclass level. Triterpenoids accounted for 45 of these 68 classified features, followed by six flavonoids, six steroids, two diterpenoids, and smaller numbers of other classes (Tables S2 and S3). Thus, triterpenoids represented approximately two thirds of the features for which a superclass could be assigned. At the class level, oleanane triterpenoids were the largest group with 27 features, and accounted for 60% of the classified triterpenoids. The remaining triterpenoid features were distributed among ursane, taraxastane, cycloartane, lupane, lanostane-related, and cucurbitane classes. The activity-tracking chemistry therefore did not correspond to a single compound series. Instead, several triterpenoid skeletons followed the same fraction-level phenotype, with oleanane-related structures forming the largest annotated group. The high number of features that remained unclassified at the superclass and class levels also means that these proportions describe only the structurally resolved part of the candidate set.

The same chemical pattern was evident at the molecular family level. Of the 64 molecular families containing at least five members, 18 reached *q* < 0.05 for at least one of the two permutation statistics, and 13 of these 18 families were dominated by terpenoids (Table 3). Six families were significantly enriched when the proportion of activity-tracking features within each family was considered. Comp_15 contained 14 features, of which 78.6% were activity tracking and 85.7% were classified as terpenoids. Comp_16 and comp_18 showed similar patterns, with terpenoids accounting for 84.2% and 93.8% of their members, respectively. In contrast, comp_2 was the largest family in the network with 375 features but contained only 13.9% activity-tracking members. This distinction is important, as a large family can contain multiple candidate features simply because it contains multiple features overall. The permutation analysis instead identifies families in which the fraction-associated pattern occurs more consistently than expected from family size alone.

Not all enriched families were terpenoid dominated. Comp_8 showed the highest mean log2 fold change (7.39) and contained 66.7% activity-tracking features, but it was dominated by shikimates and phenylpropanoids and contained only 6.7% terpenoid features. Comp_149 was also strongly associated with the active fractions, with 66.7% of its nine members classified as activity tracking, although its dominant chemical pathway remained unclassified. Comp_19 was likewise assigned predominantly to shikimates and phenylpropanoids, but only 17.6% of its members met the activity-tracking criteria. In contrast, comp_153, classified as amino acids and peptides, showed a significant shift in mean log2 fold change despite containing no activity-tracking features. These results indicate that the fraction-associated chemistry was not exclusively terpenoid and also illustrate why permutation significance was used primarily for prioritisation rather than as evidence of biological activity. The predominance of terpenoid chemistry therefore reflects the broader pattern across the enriched families rather than the absence of non-terpenoid signals.

Several additional families showed significant positive mean log2 fold changes even when the proportion of individual activity-tracking members did not reach significance. Comp_50 and comp_13, for example, consisted entirely of terpenoid features and were strongly shifted toward the active fractions. These results indicate that the activity-related signal was not restricted to a few highly ranked features. In several families, the broader group of structurally related metabolites was preferentially distributed into SGE and SGB. This family-level behaviour is particularly useful in the present four-fraction design because it shifts the interpretation from individual correlations with limited statistical resolution to coordinated changes within chemically related groups.

The mass and chromatographic behaviour of many enriched terpenoid families were consistent with glycosylated triterpenoids rather than free aglycones. These families were concentrated mainly between *m*/*z* 780 and 1170, and eluted between approximately 5.5 and 7.0 min. Comp_17, for example, contained 21 features eluting within a narrow retention window around 6.80 min, and included several features assigned to oleanane triterpenoids. Together with the common pentacyclic skeleton and aglycone ions described in Section 3.2, the combination of a high precursor mass, close chromatographic behaviour, and sugar-sized mass differences is consistent with a series of related triterpenoid glycosides. Similar LC-MS and molecular networking strategies have been used to resolve structurally diverse triterpenoid saponin series in Camellia japonica, where molecular networking and MS2LDA supported the prediction of 38 triterpenoid saponins before targeted isolation [38]. The family annotations nevertheless remain tentative. For some features in this high-*m*/*z* region, the charge state could not be assigned unambiguously. Accordingly, the observed *m*/*z* values are reported as spectral features rather than interpreted directly as neutral molecular masses, and precise molecular formulas or glycoside structures remain tentative. More generally, clustering with an oleanane-related family supports structural relatedness but does not establish the precise aglycone, sugar sequence, linkage position, or stereochemistry of an individual feature. These limitations are consistent with the annotation levels defined in Section 3.2.

MS2LDA provided a complementary structural view of the activity-tracking chemistry. Of 300 Mass2Motifs, 141 were tested and nine were significantly enriched after false discovery rate correction (Table 4, Figure 7A). Six were dominated by triterpenoids. Pos_motif_175 contained the characteristic pentacyclic triterpenoid ions at *m*/*z* 189.16, 191.18, 203.18, 205.20, and 249.19, while several other motifs also contained aglycone-related ions at *m*/*z* 439.36 and 455.35 (Figure 7B). Pos_motif_81 showed the strongest enrichment, with 10 of 13 members classified as activity-tracking features. The enriched motifs also included a smaller flavonoid signal. Pos_motif_123 and pos_motif_110 contained the *m*/*z* 287.06 aglycone ion, and pos_motif_110 additionally showed a 162.05 Da neutral loss, consistent with a hexose residue (Figure 7B). Pos_motif_144 contained the ions at *m*/*z* 455.35 and 191.18, which are also observed in triterpenoid-related spectra; however, none of its four member features received a terpenoid pathway assignment. Because these motif words are not by themselves sufficient for class-level identification and the motif contained only four members, pos_motif_144 was retained as structurally unresolved. These results therefore support the predominance of triterpenoid chemistry while retaining a distinct flavonoid component. Molecular networking and MS2LDA describe the same MS/MS data at different structural levels. Molecular networking groups spectra by overall similarity [14], whereas MS2LDA identifies recurring fragments and neutral losses [24,25]. Motif discovery itself was unsupervised with respect to cytotoxicity, whereas motif enrichment was evaluated using the same activity-tracking labels as the molecular family analysis. Accordingly, agreement between the two approaches reflects consistency across complementary representations of the same MS/MS dataset rather than an independent biological validation [38,39].

The two analyses converged on a substantially smaller region of the detected metabolome. A total of 334 features belonged to an enriched molecular family and also carried an enriched Mass2Motif. Of these, 80.5% were classified as terpenoids. This percentage is based on the full set of 334 features, including the 20 features (6.0%) that remained unclassified at the pathway level; the remaining classified features comprised 34 (10.2%) shikimates and phenylpropanoids and 11 (3.3%) features distributed among polyketides, amino acids and peptides, alkaloids, and fatty acids. Importantly, 102 of the original 187 activity-tracking features occurred within this overlapping set, and their median log2 fold change was 5.71, corresponding to approximately 52-fold higher abundance in the active fractions. In comparison, 2204 detected features received support from neither analysis. The 334 features should not be interpreted as 334 active compounds because family and motif membership extends beyond the features used to define the original activity-tracking set. Rather, the overlap defines a chemically coherent region around the fraction-associated features and substantially reduces the chemical space for subsequent experimental investigation (Table S4).

The predominance of triterpenoid saponin-related chemistry is also biologically plausible. A recent feature-based molecular networking study of *Xanthoceras sorbifolia* used triterpenoid molecular families to guide the isolation of barrigenol-type saponins, several of which inhibited both A549 and HepG2 cells [39]. Triterpenoid saponins isolated from *Camellia oleifera* have likewise shown markedly different antiproliferative activities depending on relatively small structural differences. In a 2025 study, some closely related saponins inhibited HepG2 proliferation, whereas analogues carrying different substitution at C16 were inactive, and the same structural difference also affected haemolytic activity [40]. These observations are relevant to the present network because structural relatedness does not imply equal biological activity. An enriched molecular family may contain active constituents together with weakly active or inactive analogues.

The combined evidence therefore supports prioritisation of glycosylated triterpenoid chemistry, particularly oleanane-related families, rather than assignment of cytotoxicity to a specific compound. The pathway classification, molecular family enrichment, and Mass2Motif analysis all point toward the same broad chemical region, while a smaller flavonoid glycoside signal is retained in parallel. This convergence provides a stronger basis for candidate selection than any individual feature from the four-fraction comparison, but it remains an association between chemical distribution and the observed cellular phenotype. Direct evidence for the contributions to cytotoxicity requires isolation and testing of individual members of the prioritised families.

### 3.5. Isolation and Cytotoxicity of Pure Constituents

To provide such evidence, SGE showed the lowest IC_50_ values against both cell lines and was therefore selected for chromatographic separation (Figure 8); SGB, the other active fraction, was not taken forward for parallel isolation in the present study given the scale of the chromatographic work involved, yielding a series of subfractions and ten isolated compounds. Of these ten compounds, nine were structurally confirmed by HR-MS and 1D/2D NMR, while daucosterol was assigned by chromatographic comparison with an authentic standard.

The ten isolated constituents comprised five flavonoids—two flavones, apigenin (SGE11) [41] and 4′,7-dihydroxyflavone (SGE14) [42]; one isoflavone, formononetin (SGE6.4) [43]; one flavonol, kaempferol (SGE10) [44]; and one chalcone, isoliquiritigenin (SGE9.5.1) [45]—as well as two oleanane-type triterpenoids, oleanolic acid (SGE5) [46] and olean-12-ene-1,3,23-triol (SGE8.1) [47]; one oleanane-type diglycoside, 3-O-α-L-rhamnopyranosyl-(1→3)-β-D-glucuronopyranosyl oleanolic acid (SGE20) [48]; one phytosterol, daucosterol (SGE17) [49]; and one furan derivative, 5-hydroxymethylfurfural (SGE7.2.2) [50]. Their NMR and MS data are provided in Tables S6–S14 and Figure S1. Among them, the diglycoside SGE20 has previously been reported from the aerial parts of *Sesbania sesban*, where it was associated with molluscicidal activity [48]. Its isolation here indicates that the same saponin also occurs in another species of the genus. The isolation of 5-hydroxymethylfurfural (5-HMF; SGE7.2.2) may also be relevant to post-harvest processing. Because 5-HMF can form during the Maillard reaction and caramelisation and increases during storage under suitable temperature and water-activity conditions [50], its presence may be reflected in drying, storage, extraction, or concentration. Accordingly, 5-HMF is considered a potential process-related constituent rather than a confirmed native component of fresh flowers.

The isolated compounds and subfractions showed different levels of cytotoxicity against A549 and HepG2 cells (Figure 8). SGE20 was the most active tested isolate, with IC_50_ values of 33.08 µM against A549 cells and 47.54 µM against HepG2 cells. Apigenin (SGE11) showed weaker activity, with IC_50_ values of 128.56 and 163.34 µM. 4′,7-Dihydroxyflavone (SGE14) and isoliquiritigenin (SGE9.5.1) showed weaker activity still, with IC50 values of 278.72 µM against A549 cells and 243.69 µM against HepG2 cells for SGE14, and 280.74 µM against A549 cells and 256.57 µM against HepG2 cells for SGE9.5.1; both estimates fall within the tested concentration range (up to 786.66 µM and 780.49 µM) and are therefore interpolated rather than extrapolated values. Formononetin (SGE6.4) and olean-12-ene-1,3,23-triol (SGE8.1) were inactive at the highest tested concentration (200 µg/mL, corresponding to 745.55 µM and 436.00 µM, respectively) against both cell lines. Oleanolic acid (SGE5), kaempferol (SGE10), and daucosterol (SGE17) were not evaluated in the present assay because the quantities isolated were insufficient. For the same reason, no additional SGE20 remained for spike-in or selective-removal validation in the intact SGE fraction or its subfractions. Ellipticine, used as the positive control, gave IC_50_ values of 1.58 µM against A549 cells and 1.42 µM against HepG2 cells.

SGE20 was assigned to feature 2512 in the aligned metabolomic dataset (*m*/*z* 779.4564, 7.33 min). The observed precursor matched the protonated molecule of C42H66O13, the molecular formula established by NMR, with a mass error of 1.6 ppm relative to the calculated value of 779.4576. Its reconstructed MS/MS spectrum contained characteristic oleanane-related ions at *m*/*z* 189.17, 191.18, and 203.18, together with an aglycone ion at *m*/*z* 439.36, consistent with dehydrated oleanolic acid, and an ion at *m*/*z* 585.42 retaining one deoxyhexose residue. The 340.10 Da difference between the precursor and aglycone ions was consistent with the combined loss of rhamnosyl and glucuronosyl residues together with water, in agreement with the 3-O-[α-L-rhamnopyranosyl-(1→3)-β-D-glucuronopyranosyl] substitution established by NMR. SGE20 itself was identified at Level 1 through HR-MS and NMR. The link between the purified compound and feature 2512 is supported by accurate mass and fragmentation behaviour. Although the purified compound was analysed using the same UHPLC-QTOF method as the metabolomic samples, a direct co-injection experiment was not performed; therefore, the correspondence is reported as provisional [19]. Definitive confirmation would require retention-time matching and co-injection under identical chromatographic conditions, which is planned as follow-up work. The main fragment ions of feature 2512 were also represented in pos_motif_73 and pos_motif_69, two of the nine Mass2Motifs enriched among the activity-associated chemistry. Feature 2512 belonged to comp_2, one of the 18 enriched molecular families, and was among the 334 features supported by both the family-level and substructure-level analyses (Table S4). It was not included among the 187 activity-tracking features because its abundance was high in SGE but not in SGB, where it was comparable with the inactive fractions. The feature-level criterion was specifically designed to identify compounds whose abundance followed the pattern of both active fractions, whereas feature 2512 represented chemistry enriched mainly in SGE. Thus, the isolation of SGE20 complements the cross-fraction analysis by identifying an additional cytotoxic constituent within SGE. Importantly, the same chemical region was still captured by the family-level and Mass2Motif analyses. Kaempferol was also matched to an activity-tracking feature by accurate mass and retention time. However, because kaempferol was not tested for cytotoxicity in the present study, this match provides evidence of chemical correspondence only.

Overall, the isolation results supported the chemical interpretation obtained from the metabolomic workflow. Representatives of both the triterpenoid and flavonoid classes highlighted by the MS-based analyses were isolated from SGE and confirmed by NMR. SGE20 was the most active tested isolate, whereas the isolated flavonoids and olean-12-ene-1,3,23-triol were inactive or only weakly active at the concentrations tested. Oleanolic acid (SGE5), the corresponding free aglycone, was not evaluated, so a direct comparison between the glycoside and aglycone cannot be made from the present data. Previous studies nevertheless provide a possible structural explanation for the activity of SGE20. Oleanolic acid monodesmosides containing glucuronic acid at C-3 have been reported to show greater cytotoxicity than related bidesmosides or oleanolic acid itself [51]. More broadly, a free carboxyl group at C-28, together with a sugar chain at C-3, has been associated with cytotoxic activity in triterpene saponins [52]. SGE20 contains both structural features, providing a plausible explanation for its activity, although this relationship was not tested directly here. The isolation of SGE20 therefore provides structural and preliminary biological support for the triterpenoid chemistry prioritised by the family-level and Mass2Motif analyses. However, it does not demonstrate that these molecular families alone account for the cytotoxicity of the whole SGE fraction. The absolute content of SGE20 in SGE was not quantified because the untargeted LC–MS analysis did not include compound-specific calibration. Therefore, whether SGE20 was present at a sufficient concentration to account for the IC_50_ of SGE could not be determined. Several higher-mass polyglycosylated members of the enriched families, eluting at approximately 5.5–7.0 min, were not obtained in a pure form and could not be tested individually. Their contribution to the activity of SGE therefore remains to be determined. The different activities observed among the isolated compounds also show that cytotoxicity is not uniformly distributed among constituents of the same fraction. These results support the metabolomic prioritisation while highlighting the remaining enriched triterpenoid members as suitable targets for further purification and direct bioactivity testing. The use of a single shade-drying and extraction protocol did not allow native flower constituents to be distinguished from processing-related products. Drying, extraction, concentration, and storage may also have affected heat-sensitive, oxidation-sensitive, or glycoside-hydrolysis-sensitive metabolites, including the prioritised saponins. Comparative analysis of prospectively matched fresh, freeze-dried, shade-dried, and cooked samples, together with targeted stability studies, is therefore required to determine the effects of processing on these constituents.

## 4. Conclusions

Taken together, the study progressed from fraction-level cytotoxicity to chemical prioritisation and experimental isolation. The MTT assay identified SGE and SGB as the fractions with the lowest IC_50_ values under the experimental conditions, while feature-level analysis further narrowed the associated chemistry to 187 activity-tracking signals. Chemical classification, molecular family enrichment, and complementary MS2LDA analyses consistently highlighted glycosylated triterpenoid-related chemistry, together with a smaller flavonoid component. Isolation of one NMR-confirmed oleanane diglycoside with moderate MTT activity provided preliminary experimental support for prioritisation, and SGE20 was the most active tested isolate, in agreement with the prioritised chemistry. The corresponding feature belonged to an enriched molecular family and carried enriched Mass2Motifs, although it was not retained by the feature-level ranking because its abundance was largely restricted to one of the two active fractions. This result shows that the family-level and substructure-level analyses provided complementary coverage beyond the four-fraction ranking alone. The main outcome is therefore not the attribution of activity to a single compound, but the identification of specific molecular families associated with the observed cellular phenotype and suitable for further targeted isolation and biological evaluation. As reported throughout, these family- and substructure-level analyses were used for candidate prioritisation rather than as confirmatory statistical inference. Overall, the study prioritises terpenoid-enriched molecular families and glycosylated triterpenoid-related substructures as targets for further isolation, re-injection-based annotation, and direct bioactivity testing. Because *S. grandiflora* flowers are generally consumed after cooking, the present findings from a shade-dried ethanolic extract should not be directly extrapolated to cooked-food composition, dietary exposure, or food safety. The present study did not evaluate the effects of cooking, realistic dietary intake, digestion, absorption, bioavailability, intestinal metabolic transformation, toxicity to normal tissues, or long-term exposure. Future studies should examine these factors under food-relevant processing and exposure conditions.

## Figures and Tables

**Figure 1:**
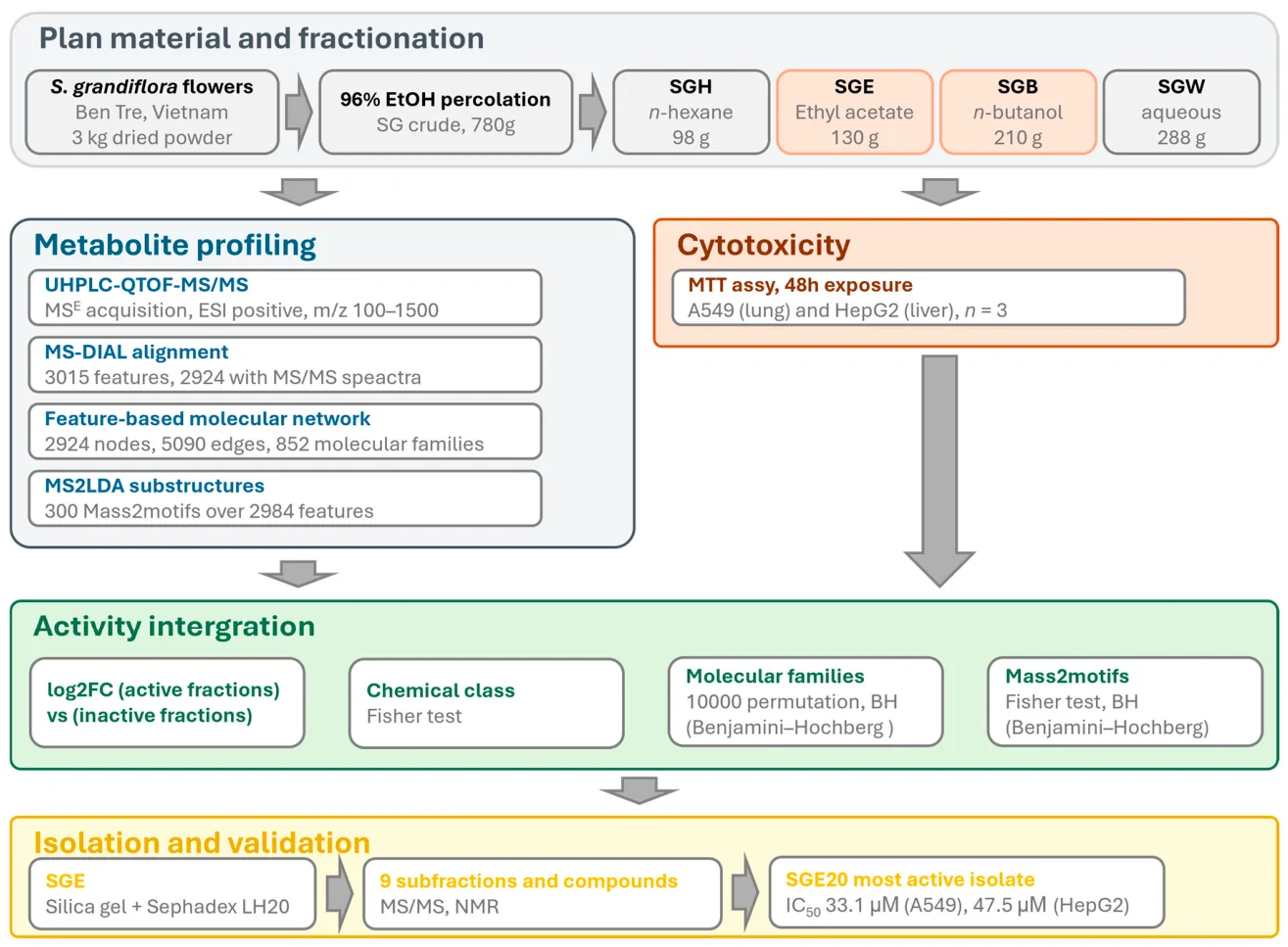
Extraction and solvent partitioning of *S. grandiflora* flowers workflow.

**Figure 2:**
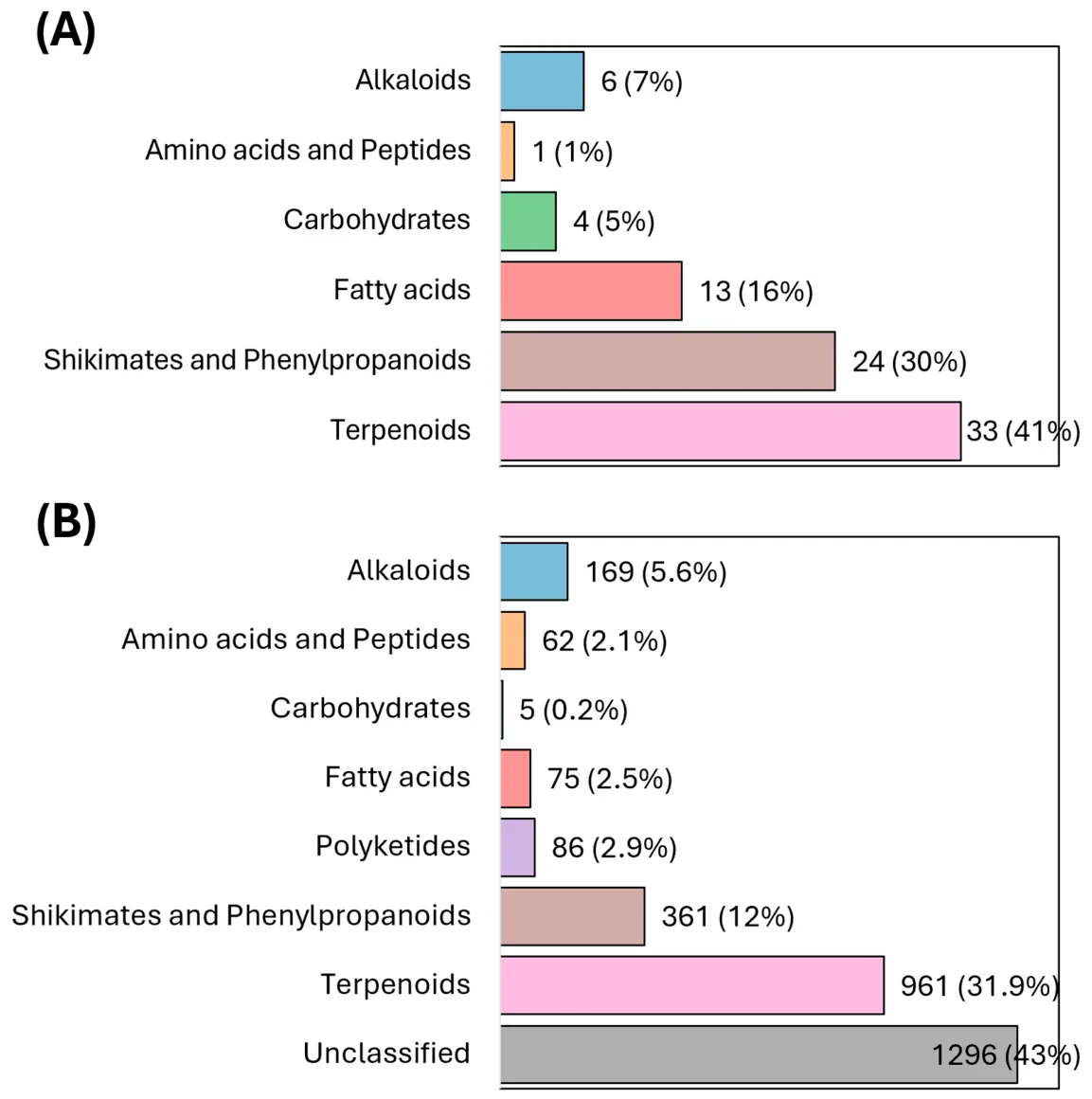
(**A**) NPClassifier pathway distribution for the 81 *Sesbania* compounds recorded in Reaxys. (**B**) NPClassifier pathway distribution for all 3015 detected features.

**Figure 3:**
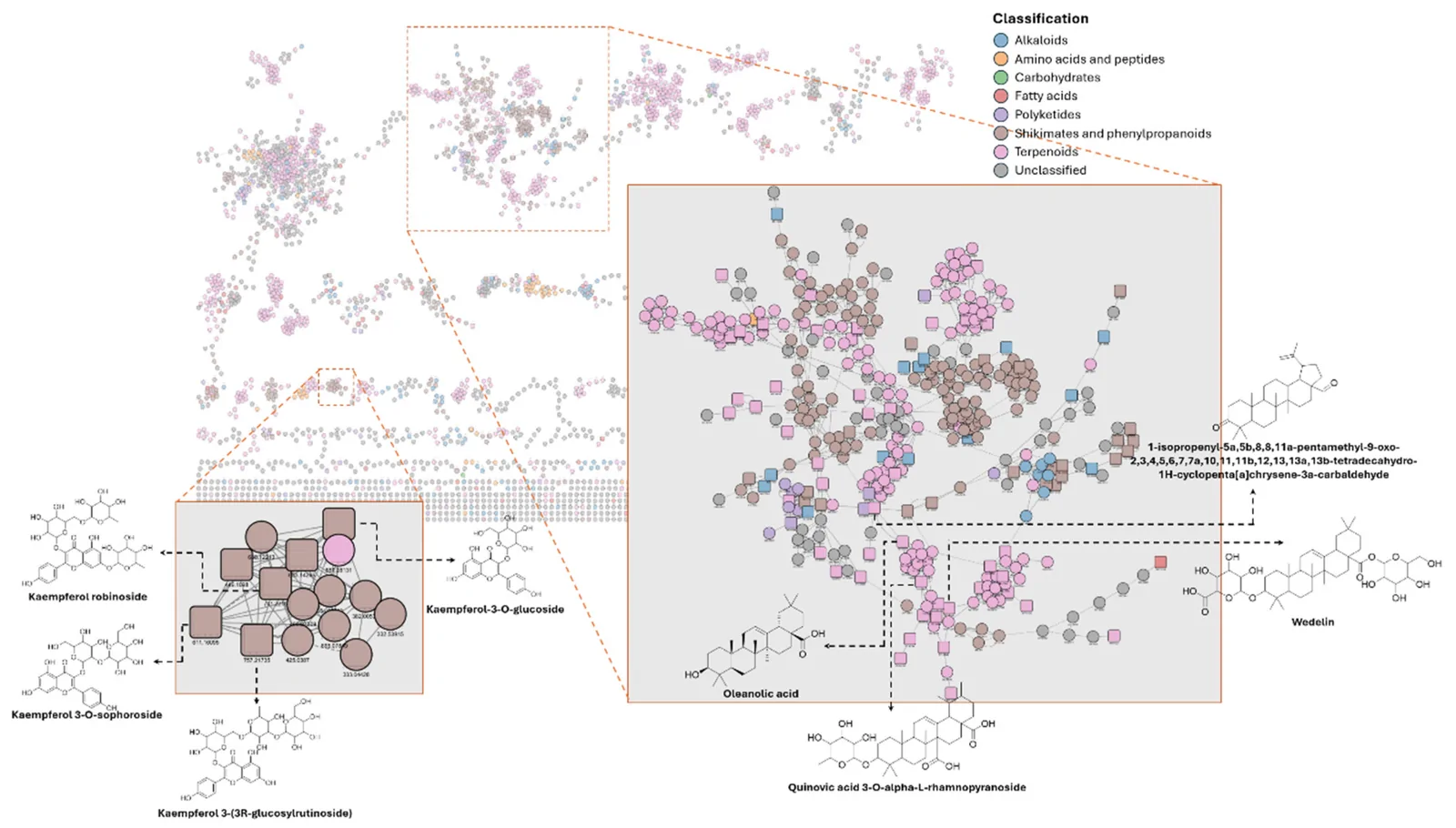
Feature-based molecular network of the four fractions (2924 nodes, 5090 edges), with nodes coloured by their NPClassifier pathway. Families discussed in the text are labelled.

**Figure 4:**
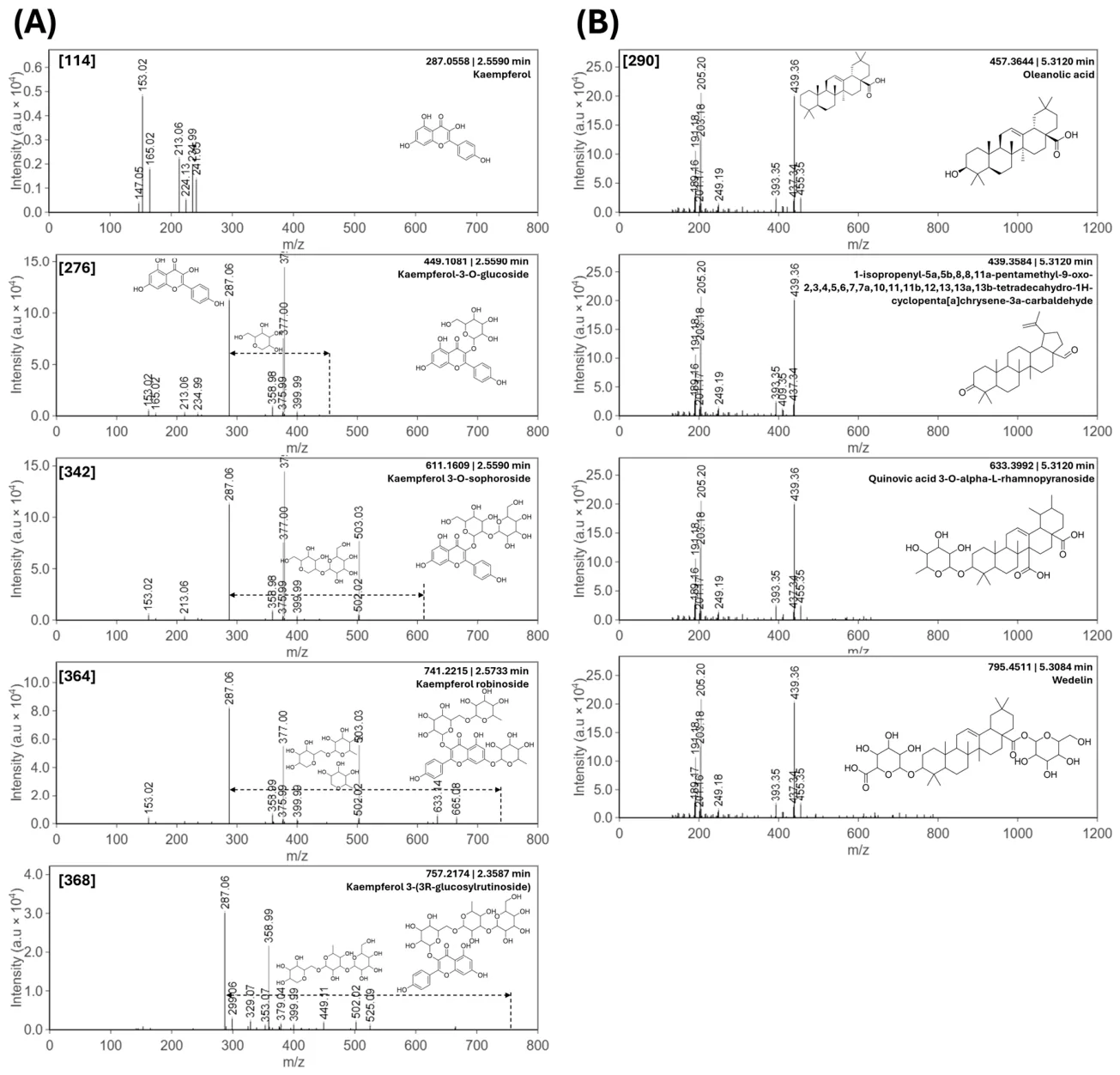
Representative MS/MS spectra of the two dominant chemistries, with tentative annotations. (**A**) Flavonol series comprising the kaempferol aglycone (*m*/*z* 287.06) and three glycosylated features differing by hexose losses, all retaining the aglycone ion as the base peak, with secondary ions at *m*/*z* 153.02, 165.02, and 213.06. (**B**) Oleanane-type triterpenoid series showing shared skeletal fragment ions at *m*/*z* 189.16, 191.18, 203.18, 205.20, and 249.19, together with aglycone ions at *m*/*z* 439.36, 437.34, and 455.35.

**Figure 5:**
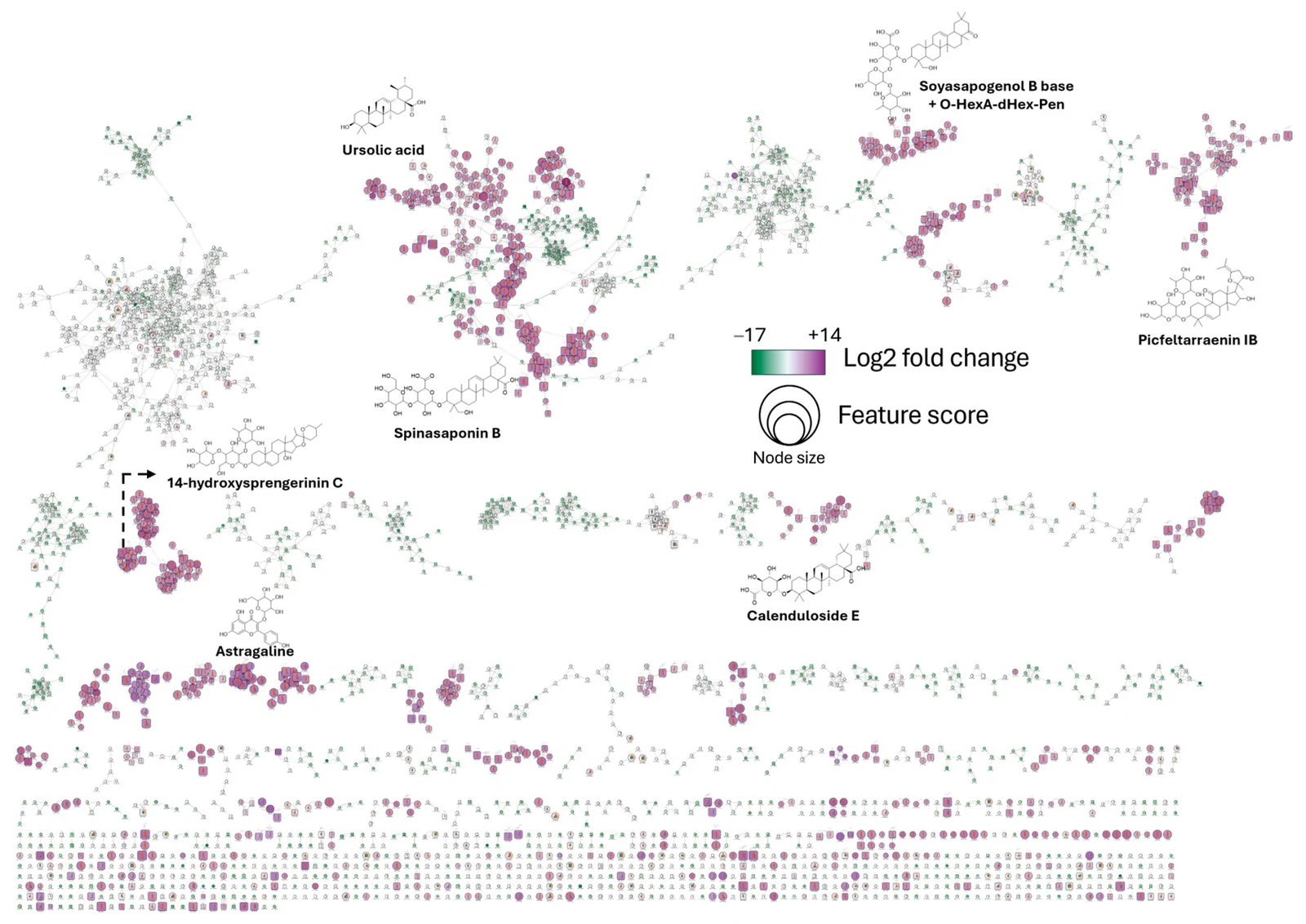
Activity association projected onto the consensus molecular network. Nodes are the 2924 features carrying MS/MS spectra and edges connect spectrally similar features. Node colour shows the log2 fold change in intensity between active fractions (SGE and SGB) and inactive fractions (SGH and SGW). Node size shows the composite feature score, which combines the Spearman coefficient against potency with a capped fold change.

**Figure 6:**
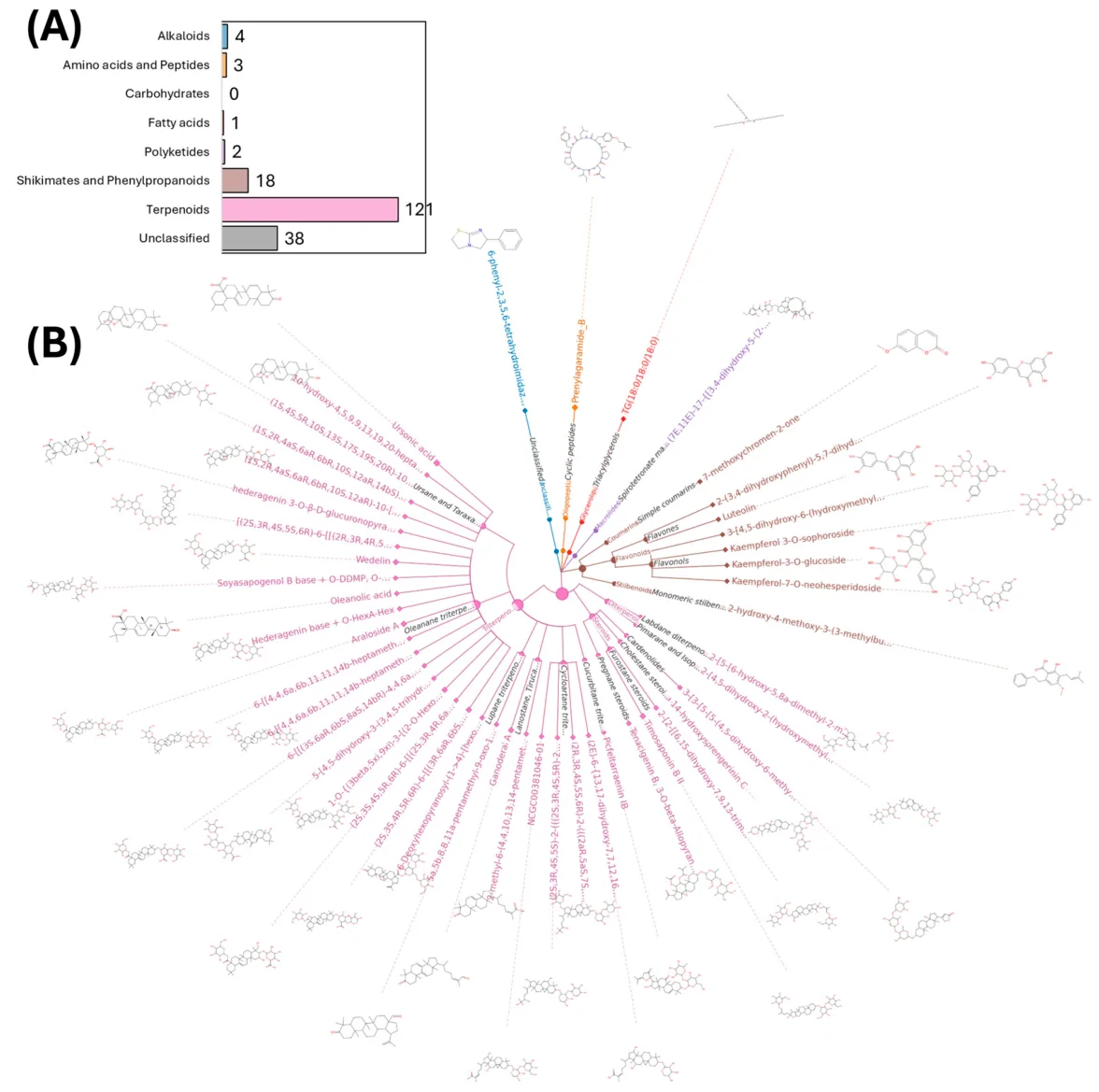
Chemical classification and annotation of the activity-tracking features. (**A**) NPClassifier pathway distribution of the 187 activity-tracking features identified across the four solvent fractions. Numbers indicate the number of features assigned to each chemical pathway. (**B**) Hierarchical representation of the activity-tracking features for which compound-level annotations were available, organised according to their assigned chemical classes and annotated structures.

**Figure 7:**
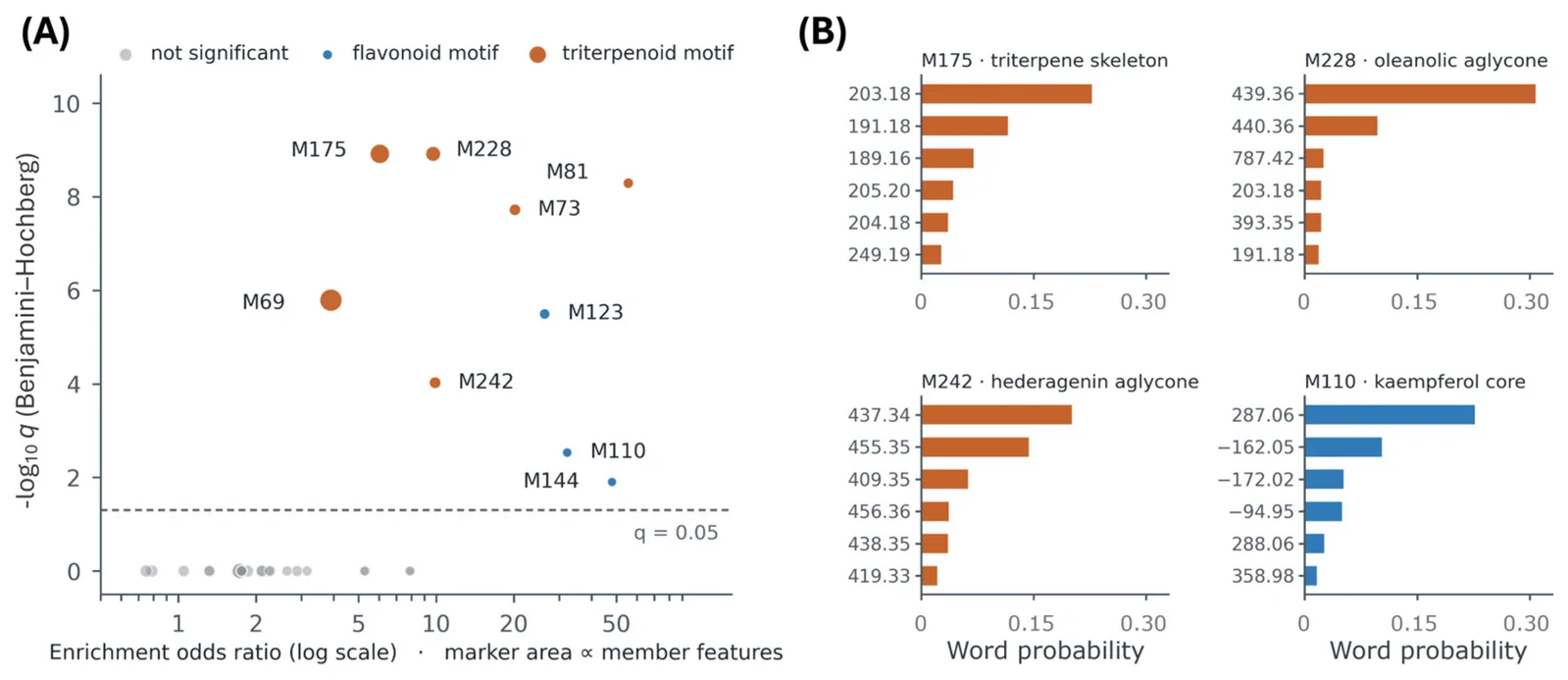
Mass2Motifs enriched among activity-tracking features. (**A**) Enrichment odds ratio and Benjamini–Hochberg *q* values for the 141 tested motifs. (**B**) Highest probability fragment and neutral loss words for four representative enriched motifs, showing diagnostic triterpene skeleton-, oleanolic acid-, hederagenin-, and kaempferol-related signals.

**Figure 8:**
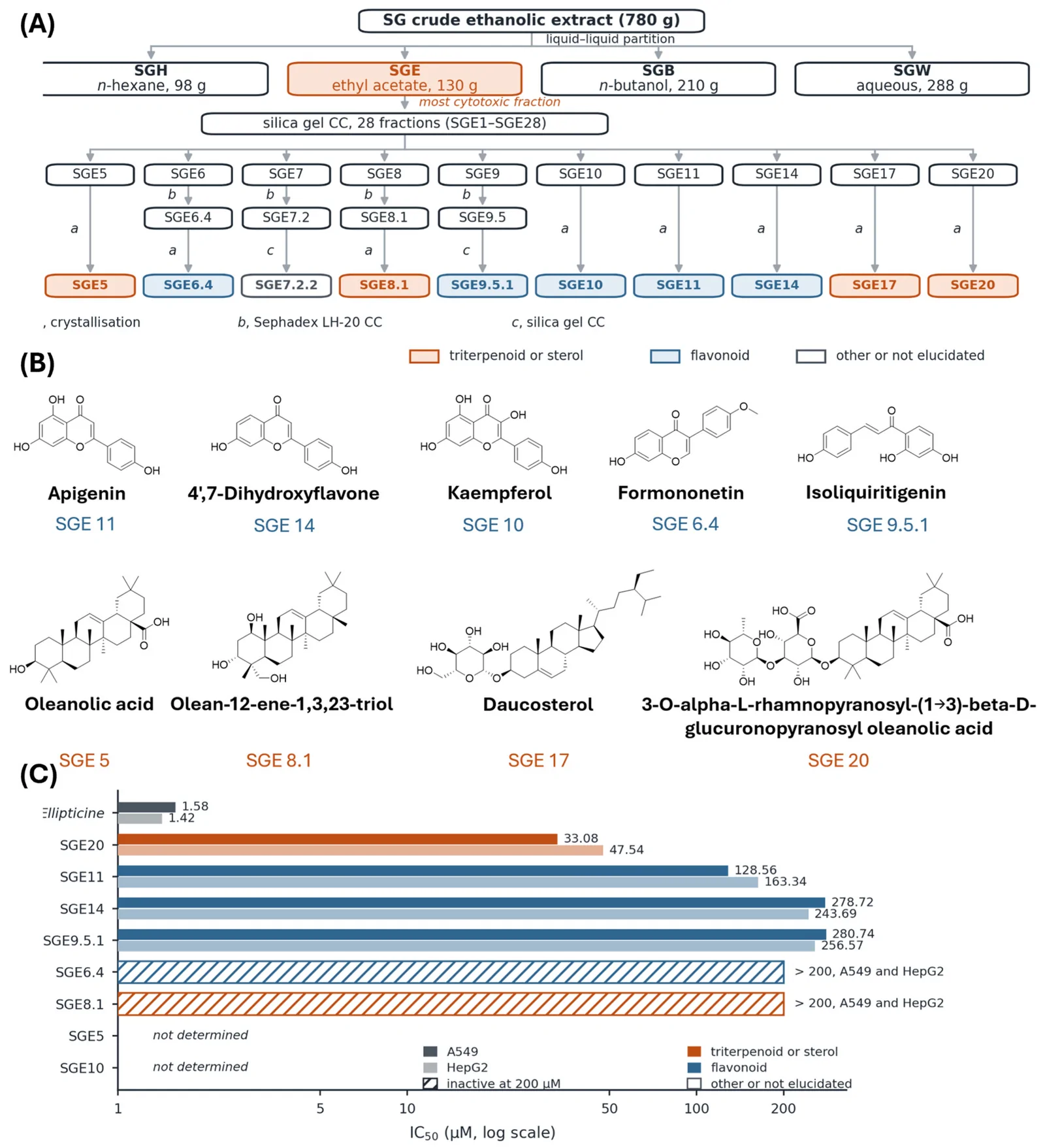
Isolation and cytotoxicity of SGE constituents. (**A**) Fractionation scheme: *a*, crystallisation; *b*, Sephadex LH-20; and *c*, silica gel chromatography. Colours indicate chemical class: orange, triterpenoid/sterol; blue, flavonoid; and white, unidentified. (**B**) Structures confirmed by HR-MS and NMR. (**C**) IC_50_ values against A549 and HepG2 cells (three independent replicates, logarithmic scale). Hatched bars indicate inactivity at the highest concentration tested (200 µg/mL); because the isolated compounds differed in molecular weight, this corresponded to a compound-specific molar concentration of 257–787 µM rather than a uniform 200 µM cutoff (Section 2.8). Values above this concentration are extrapolated. Ellipticine was used as a positive control in a separate assay run.

**Table 1:** Cytotoxicity of the fractions against A549 and HepG2 cells (IC_50_, µg/mL; mean ± SEM, *n* = 3).

Sample	A549	HepG2
SGH (*n*-hexane fraction)	>100	>100
SGE (ethyl acetate fraction)	30.44 ± 1.18	27.66 ± 1.42
SGB (*n*-butanol fraction)	31.17 ± 1.46	42.44 ± 2.62
SGW (aqueous fraction)	>100	>100
Ellipticine *	0.37 ± 0.02	0.31 ± 0.02

* Positive control, tested at 10, 2, 0.4, and 0.08 µg/mL.

**Table 2:** Distribution of NPClassifier pathways across all 3015 features and among the 187 activity-tracking features, with enrichment statistics (using one-sided Fisher exact test).

Pathway	Activity-Tracking Features (*n*)	All Features (*n*)	% of Activity-Tracking Features	% of All Features	Odds Ratio	*p*	q (BH)
Terpenoids	121	961	64.7	31.9	4.34	1.6 × 10^−21^	1.3 × 10^−20^
Unclassified	38	1296	20.3	43.0	0.32	n.s.	n.s.
Shikimates and phenylpropanoids	18	361	9.6	12.0	0.77	n.s.	n.s.
Alkaloids	4	169	2.1	5.6	0.35	n.s.	n.s.
Amino acids and peptides	3	62	1.6	2.1	0.77	n.s.	n.s.
Polyketides	2	86	1.1	2.9	0.35	n.s.	n.s.
Fatty acids	1	75	0.5	2.5	0.20	n.s.	n.s.
Carbohydrates	0	5	0.0	0.2	0.00	n.s.	n.s.
Total	187	3015	100	100			

n.s., not significant after Benjamini–Hochberg correction.

**Table 3:** Molecular families of the consensus network enriched in activity-tracking features (10,000 permutations, Benjamini–Hochberg correction across the 64 families with *n* ≥ 5). Families reaching q < 0.05 on at least one of the two statistics are listed.

Family	Features (*n*)	Mean log_2_FC	Activity-Tracking Features (%)	*q* (Mean log_2_FC)	*q* (Proportion)	Dominant Pathway	Terpenoids (%)
comp_8	15	7.39	66.7	4.6 × 10^−4^	1.1 × 10^−3^	Shikimates and phenylpropanoids	6.7
comp_149	9	6.82	66.7	4.6 × 10^−4^	1.1 × 10^−3^	Unclassified	22.2
comp_15	14	6.55	78.6	4.6 × 10^−4^	1.1 × 10^−3^	Terpenoids	85.7
comp_18	64	6.00	25.0	4.6 × 10^−4^	1.1 × 10^−3^	Terpenoids	93.8
comp_16	19	4.71	42.1	4.6 × 10^−4^	1.1 × 10^−3^	Terpenoids	84.2
comp_2	375	1.05	13.9	4.6 × 10^−4^	1.1 × 10^−3^	Terpenoids	42.1
comp_43	17	6.15	23.5	4.6 × 10^−4^	0.142	Terpenoids	88.2
comp_1	100	2.49	12.0	4.6 × 10^−4^	0.142	Terpenoids	74.0
comp_240	5	6.73	40.0	1.3 × 10^−3^	0.228	Unclassified	40.0
comp_19	17	6.35	17.6	4.6 × 10^−4^	0.527	Shikimates and phenylpropanoids	29.4
comp_17	21	4.67	14.3	4.6 × 10^−4^	0.726	Terpenoids	85.7
comp_10	69	4.23	10.1	4.6 × 10^−4^	0.726	Terpenoids	85.5
comp_50	7	6.88	14.3	4.6 × 10^−4^	n.s.	Terpenoids	100
comp_14	15	5.09	13.3	4.6 × 10^−4^	n.s.	Terpenoids	53.3
comp_13	14	5.08	7.1	4.6 × 10^−4^	n.s.	Terpenoids	100
comp_153	5	5.86	0.0	2.0 × 10^−3^	n.s.	Amino acids and peptides	0
comp_44	5	5.54	20.0	3.4 × 10^−3^	n.s.	Terpenoids	100
comp_12	10	3.42	0.0	1.0 × 10^−2^	n.s.	Terpenoids	90.0

Note: Identical *q* values occur when multiple molecular families reach the minimum attainable empirical *p* value of 1/10,001 in the 10,000-permutation test, and remain tied after Benjamini–Hochberg correction.

**Table 4:** Mass2Motifs enriched among activity-tracking features (one-sided Fisher exact test, Benjamini–Hochberg correction across 141 motifs with *n* ≥ 3), with the highest-probability motif words.

Motif	Features (*n*)	Activity-Tracking Features (%)	Odds Ratio	*q*	Median log_2_FC	Terpenoids (%)	Principal Motif Words
pos_motif_228	56	35.7	9.7	1.2 × 10^−9^	6.01	98	439.36, 440.36, 787.42, 203.18, 393.35
pos_motif_175	112	25.0	6.1	1.2 × 10^−9^	5.25	62	203.18, 191.18, 189.16, 205.20, 249.19
pos_motif_81	13	76.9	55.6	5.1 × 10^−9^	6.36	100	453.34, 435.33, 407.33, 471.35, 268.27
pos_motif_73	22	54.5	20.2	1.9 × 10^−8^	5.71	100	439.36, 203.18, 191.18, 189.17, 393.35
pos_motif_69	156	17.9	3.9	1.6 × 10^−6^	5.68	88	439.36, 203.18, 293.12, 191.18, 393.35
pos_motif_123	13	61.5	26.4	3.2 × 10^−6^	7.52	8	287.06, 299.09, 317.21, 349.15
pos_motif_242	24	37.5	9.9	9.5 × 10^−5^	6.06	83	437.34, 455.35, 409.35, 419.33
pos_motif_110	6	66.7	32.2	3.0 × 10^−3^	6.09	0	287.06, loss 162.05, loss 172.02
pos_motif_144 *	4	75.0	48.1	1.3 × 10^−2^	6.98	0	loss 178.01, 455.35, 191.18

* Terpenoid proportion is based on NPClassifier pathway assignments of motif members. Pos_motif_144 contained triterpenoid-related ions, but none of its four members were classified as a terpenoid, and the motif was therefore retained as unresolved.

## Data Availability

The original contributions presented in this study are included in the article/Supplementary Materials. Further inquiries can be directed to the corresponding author.

## Supplementary Materials

The following supporting information can be downloaded at: https://www.mdpi.com/article/10.3390/foods15193455/s1, Table S1 contains the annotated features with the adduct, retention time, observed and theoretical *m*/*z*, mass error, chemical class, and annotation confidence, as well as the ten most intense diagnostic fragment ions. Tables S2 and S3 contain the 187 activity-tracking features identified from the A549 and HepG2 cytotoxicity assays, respectively. Table S4 lists the 334 features supported by both the molecular family and Mass2Motif analyses. Table S5 summarises the 64 molecular families included in the family-level enrichment analysis. Tables S6–S14 provide the NMR data of the isolated compounds.
